# Fibroblasts derived from long-lived insulin receptor substrate 1 null mice are not resistant to multiple forms of stress

**DOI:** 10.1111/acel.12255

**Published:** 2014-07-24

**Authors:** Melissa M Page, Amy Sinclair, Ellen L Robb, Jeffrey A Stuart, Dominic J Withers, Colin Selman

**Affiliations:** 1 Integrative and Environmental Physiology, Institute of Biology and Environmental Sciences, University of AberdeenAberdeen, AB24 2TZ, UK; 2 Institute of Biodiversity, Animal Health and Comparative Medicine, College of Medicine, Veterinary and Life Sciences, Graham Kerr Building, University of GlasgowGlasgow, G12 8QQ, UK; 3 Department of Biological Sciences and Cold Climate Oenology and Viticulture Institute, Brock UniversitySt. Catharines, ON, L2S 3A1, Canada; 4 Metabolic Signaling Group, Medical Research Council Clinical Sciences Centre, Imperial CollegeLondon, W12 0NN, UK; * Department of Cellular and Physiological Sciences, University of British ColumbiaVancouver, BC, V6T 1Z3, Canada; † MRC Mitochondrial Biology Unit, Mitochondrial DysfunctionWellcome Trust/MRC Building, Hills Road, Cambridge, CB2 0XY, UK

**Keywords:** aging, IRS1, NRF2, oxidative stress, stress resistance

## Abstract

Reduced signalling through the insulin/insulin-like growth factor-1 signalling (IIS) pathway is a highly conserved lifespan determinant in model organisms. The precise mechanism underlying the effects of the IIS on lifespan and health is currently unclear, although cellular stress resistance may be important. We have previously demonstrated that mice globally lacking insulin receptor substrate 1 (*Irs1*^*−/−*^) are long-lived and enjoy a greater period of their life free from age-related pathology compared with wild-type (WT) controls. In this study, we show that primary dermal fibroblasts and primary myoblasts derived from *Irs1*^*−/−*^ mice are no more resistant to a range of oxidant and nonoxidant chemical stressors than cells derived from WT mice.

## Introduction

It is evident that the insulin/insulin-like growth factor-1 signalling (IIS) pathway plays a conserved role in lifespan determination (Gems & Partridge, 2013). Precisely how the IIS elicits its effects is unclear, although stress resistance in longevity may be important. Several long-lived IIS mutant worms and flies are stress resistant compared with wild-type (WT) controls [see (Miller, 2009)], and stress resistance correlates with longevity across species of mammals (Kapahi *et al*., 1999; Harper *et al*., 2007) and birds (Harper *et al*., 2011). An increased survival following paraquat or diquat injection has been reported in long-lived growth hormone (GH)-deficient Ames mice (Bokov *et al*., 2009), which have a secondary defect in IIS, and in female mice lacking a single copy of the IGF-1 receptor (Igf1R^+/−^) (Holzenberger *et al*., 2003; Bokov *et al*., 2011). A significant body of work has described broad-spectrum cellular resistance to various oxidative and nonoxidative stressors in GH dwarfs (Murakami *et al*., 2003; Salmon *et al*., 2005; Miller, 2009). While the precise mechanism underlying cellular stress resistance in GH dwarfs is unclear, it is associated with increased basal and arsenite-induced Nrf2 levels (Leiser & Miller, 2010), increased autophagy and reduced mTOR activity (Wang & Miller, 2012).

**Figure 1:**
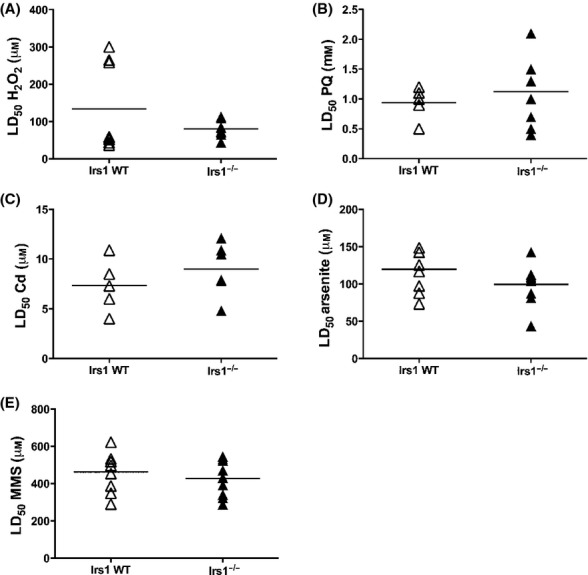
Fibroblasts from *Irs1*^*−/−*^ mice are not more resistant to lethal stress than fibroblasts derived from WT mice at 21% O_2_. Each symbol represents fibroblasts derived from a single individual; the horizontal line indicates mean value for each group (*n* = 7–9). (A) H_2_O_2_, (B) paraquat, (C) cadmium, (D) arsenite and (E) MMS. *denotes *P* < 0.05. The test statistics and p values are reported in Table S1.

**Figure 2:**
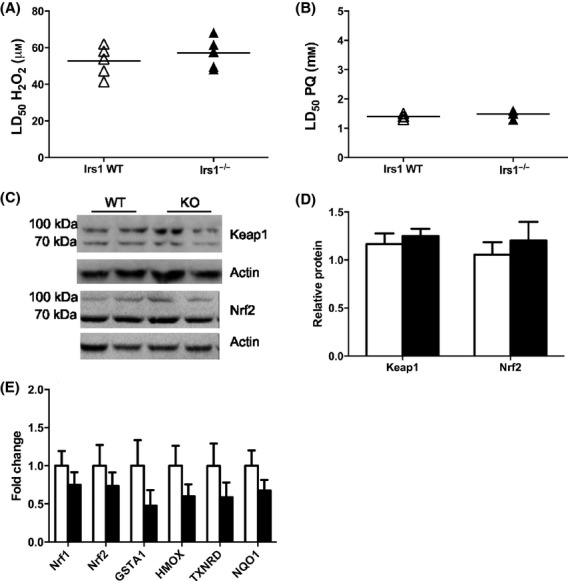
Myoblasts from *Irs1*^*−/−*^ mice are not more resistant to (A) H_2_O_2_ and (B) paraquat lethal stress than myoblasts from WT mice at 3% O_2_, and basal protein levels of Keap1 and Nrf2 (C, D) and basal transcript levels of Nrf1, Nrf2 and associated targets in fibroblasts are unaltered in *Irs1*^*−/−*^ mice relative to WT mice. Each symbol in (A) and (B) represents myoblasts derived from a single individual; the horizontal line indicates mean value for each group (*n* = 6). The test statistics and p values are reported in Table S2. (C) Representative western blots of Keap1 and Nrf2 from fibroblasts, (D) quantification of Keap1 and Nrf2 protein levels and (E) transcript levels of *Nrf-1, Nrf-2*, glutathione S-transferase, alpha 1 (*GSTA1*), haem oxygenase-1 (*HMOX*), thioredoxin-1 (*TXNRD*), NAD(P)H quinone oxidoreductase 1 (*NQO1*) in fibroblasts from WT and *Irs1*^*−/−*^ mice (*n* = 6) cultured at 21% O_2_, all target genes are corrected for the expression of *Gapdh* and *Hprt*. Data are normalized to WT values in all cases, *P* > 0.05. Mean ± SEM. Open bars = WT and filled bars = *Irs1*^*−/−*^ mice.

We previously reported that *Irs1*^*−/−*^ mice were long-lived and had improved late-life health (Selman *et al*., 2008, 2011). Consequently, we predicted that *Irs1*^*−/−*^ mice would demonstrate increased cellular stress resistance. To address this, we isolated and cultured dermal fibroblasts from young *Irs1*^*−/−*^ and WT mice using published protocols (Murakami *et al*., 2003; Salmon *et al*., 2005; Leiser & Miller, 2010). Cells were exposed to hydrogen peroxide (H_2_O_2_), paraquat, cadmium chloride, arsenite and the DNA-alkylating agent methyl methanesulfonate (MMS) under both standard (21% oxygen) and physiological (3% oxygen) conditions in parallel experiments. Cytotoxicity was determined by calculating LD_50_ using probit analysis (Leiser & Miller, 2010). In contrast to our prediction, fibroblasts from *Irs1*^*−/−*^ mice were not more resistant to cytotoxic stress at 21% oxygen (Fig. 1A–E) or 3% oxygen (Fig. S1A–E). Indeed, *Irs1*^*−/−*^ cells were significantly more sensitive when treated with arsenite at 21% O_2_ (Fig. 1D). We also exposed primary myoblasts cultured at 3% oxygen (21% induced differentiation to myotubes) to H_2_O_2_ and paraquat and observed no genotype effect on cellular resistance (Fig. 2A,B). Additionally, no differences in basal Nrf2 or Keap1 protein levels (Fig. 2C,D), or in basal levels of Nrf1, Nrf2 and various antioxidant response element-dependent mRNA transcripts (Fig. 2E) were detected between WT and *Irs1*^*−/−*^ fibroblasts. Cell size, proliferation rates and cell cycle distribution in cultured fibroblasts were similarly unaffected by genotype (Fig. S2A–C; *P* > 0.05 in all cases).

While both male and female *Irs1*^*−/−*^ mice are long-lived (Selman *et al*., 2008, 2011), we did not observe a cellular stress resistance phenotype as predicted and as reported in long-lived GH-deficient mice (Murakami *et al*., 2003; Salmon *et al*., 2005; Leiser & Miller, 2010). However, a similar absence of cellular stress resistance has been reported in long-lived methionine-restricted and caloric-restricted (CR) mice (Harper *et al*., 2006). A potential explanation given for the different cellular stress phenotypes in GH dwarfs, methionine-restricted and CR mice was that epigenetic changes leading to cellular stress resistance may be manifested during development, rather than postnatally (Harper *et al*., 2006). However, we can discount this explanation in our study as the deletion event in *Irs1*^*−/−*^ mice occurs during development. Long-lived homozygous *Chico* flies, which lack the single *Drosophila* IRS protein (Clancy *et al*., 2001), and long-lived brain-specific *Igf1R*^*+/−*^ mice (Kappeler *et al*., 2008) are also not resistant to oxidative stress. It is currently unclear why different long-lived mouse models show such differences in cellular stress resistance. *Irs1*^*−/−*^ mice, unlike GH dwarfs, appear to have relatively normal somatotropic function and GH levels (Selman *et al*., 2008) and this may help explain the absence of a stress-resistant phenotype. In Ames mice, treatment with GH shortened lifespan, decreased cellular stress resistance (Panici *et al*., 2010) and attenuated components of the cellular detoxification pathway, including glutathione transferases (Rojanathammanee *et al*., 2013). As discussed elsewhere (Harper *et al*., 2006), it may be that the precise cell type demonstrating cellular stress resistance, and hence critical to longevity, may vary across different long-lived mouse models. For example, while fibroblasts from methionine-restricted and CR mice are not stress resistant, both models, unlike Snell and GHR–KO mice, are resistant to acetaminophen (Harper *et al*., 2006). In conclusion, our findings demonstrate that fibroblasts and myoblasts from *Irs1*^*−/−*^ mice are no more stress resistant than those derived from WT mice, suggesting that cellular stress resistance does not underlie the extended healthy lifespan of *Irs1*^*−/−*^ mice.

## Supporting Information

**Table S1** LD_50_ values of dermal fibroblasts isolated from WT and *Irs1*^*−/−*^ mice exposed to lethal oxidative and non-oxidative stressors; cultured at 3% and 21% O_2_. **Table S2** LD_50_ values of myoblasts isolated from WT and *Irs1*^*−/−*^ mice exposed to lethal oxidative stressors; cultured at 3% O_2_. **Table S3** Primers used in the present study.

**Fig. S1** Fibroblasts from *Irs1*^*−/−*^ mice are not more resistant to lethal stress than fibroblasts from WT mice at 3% O_2_. **Fig. S2** Fibroblasts from *Irs1*^*−/−*^ mice do not differ in terms of (A) proliferation rates, (B) cell cycle progression or (C) growth rates after 24 h or 48 h of culture (21% O_2_) compared with cells derived from WT mice (*n* = 4 per group).

**Data S1** Experimental Procedures.
