# Supplementary material for: Fibroblasts derived from long-lived insulin receptor substrate 1 null mice are not resistant to multiple forms of stress
Source: Aging Cell. 2014 Jul 24;13(5):962–4. doi: 10.1111/acel.12255 (PMC4331740; doi:10.1111/acel.12255)
Supplement: Supplementary file 1 — Table S1 LD50 values of dermal fibroblasts isolated from WT and Irs1−/− mice exposed to lethal oxidative and non-oxidative stressors; cultured at 3% and 21% O2. Table S2 LD50 values of myoblasts isolated from WT and Irs1−/− mice exposed to lethal oxidative stressors; cultured at 3% O2. Table S3 Primers used in the present study. [file acel0013-0962-sd1.docx]

Table S1: LD_50_ values of dermal fibroblasts isolated from WT and *Irs1^-/-^* mice exposed to lethal oxidative and non-oxidative stressors; cultured at 3% and 21% O_2_.

| Treatment | WT Mean ± SEM | *Irs1^-/-^* Mean ± SEM | Replicates | Two way ANOVA  F and (p-value) |
| --- | --- | --- | --- | --- |
| 3% O_2_ |  |  |  |  |
| H_2_O_2_ | 94.1 ± 7.9 | 96.6 ± 6.9 | 8-9 | 0.848 (0.388) |
| PQ | 2.3 ± 0.4 | 2.2 ± 0.3 | 7-8 | 0.668 (0.435) |
| Cd | 8.5 ± 1.5 | 9.6 ± 2.0 | 7 | 0.271 (0.617) |
| Arsenite | 119.9 ± 7.6 | 124.1 ± 13.5 | 8 | 0.013 (0.910) |
| MMS | 547.1 ± 56.7 | 551.3 ± 63.2 | 8 | 0.001 (0.979) |
| 21% O_2_ |  |  |  |  |
| H_2_O_2_ | 133.8 ± 41.5 | 98.0 ± 18.8 | 8-9 | 0.564* |
| PQ | 0.9 ± 0.07 | 1.1 ± 0.2 | 8-9 | 0.498 (0.495) |
| Cd | 9.2 ± 2.1 | 10.4 ± 1.7 | 6-7 | 1.532 (0.251) |
| Arsenite | 119.6 ± 9.2 | 99.5 ± 9.0 | 9 | 13.400 (0.004) ** |
| MMS | 463.2 ± 34.7 | 427.8 ± 32.3 | 9 | 1.510 (0.705) |

*Data not normally distributed p-value from Kruskal-Wallis test. ** LD_50_ was significantly elevated in WT fibroblasts compared *Irs1^-/-^* fibroblasts. O_2_; oxygen, H_2_O_2_; hydrogen peroxide, PQ; paraquat, Cd; cadmium, MMS; methyl methanesulfonate.

Table S2: LD_50_ values of myoblasts isolated from WT and *Irs1^-/-^* mice exposed to lethal oxidative stressors; cultured at 3% O_2_.

| Treatment | WT Mean ± SEM | *Irs1^-/-^* Mean ± SEM | Replicates | One way ANOVA  F and (p-value) |
| --- | --- | --- | --- | --- |
| H_2_O_2_ | 52.8 ± 3.0 | 57.2 ± 3.1 | 6 | 1.816 (0.215) |
| PQ | 1.4 ± 0.03 | 1.5 ± 0.04 | 6 | 0.947 (0.359) |

Table S3: Primers used in the present study

| Gene | Forward (5’ → 3’) | Reverse (5’ → 3’) |
| --- | --- | --- |
| *Gapdh* | AAGGTGGTGAAGCAGGCATCTG | TGTAGCCGTATTCATTGTCATACCAGG |
| *Gsta1* | AGGACATGAAGGAGAGAGCCCTGATT | TCCATGGCTCTTCAACACCTTTTC |
| *Hmox-1* | TTCCTGCTCAACATTGAGCTGTTTG | GCAAGATACTGCCCCTGCAGAGAC |
| *Hprt* | GTTAAGCAGTACAGCCCCAAA | AGGGCATATCCAACAACAAACTT |
| *Nqo1* | TTTCCAGAAATGACATCACAGGTG | AAACCACTGCAATGGGAACTG |
| *Nrf1* | CTGTGGCTGATGGAGAGGTGG | ATGGGCGCAGCTTCACTG |
| *Nrf2* | GCCAGCTACTCCCAGGTTGC | CAGGGCAAGCGACTCATGG |
| *Txnrd1* | TGGAAAGTCGAAGACACAGTGAAGC | TTCTTTACCTTTGTTATTTGTCGCCAC |
