# Supplementary figures and images for: Fibroblasts derived from long-lived insulin receptor substrate 1 null mice are not resistant to multiple forms of stress
Source: Aging Cell. 2014 Jul 24;13(5):962–4. doi: 10.1111/acel.12255 (PMC4331740; doi:10.1111/acel.12255)

Figure S1


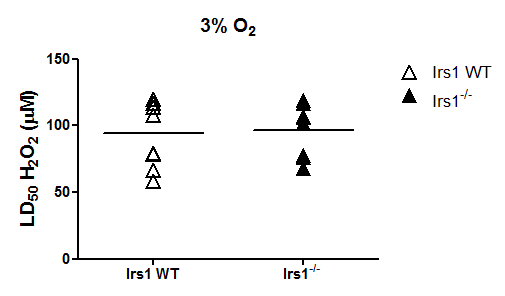

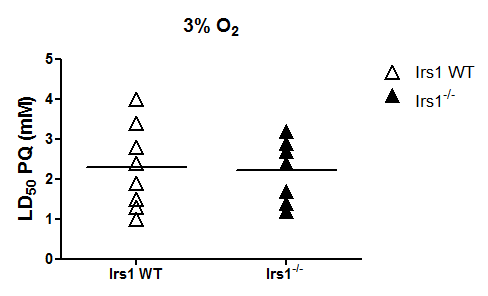

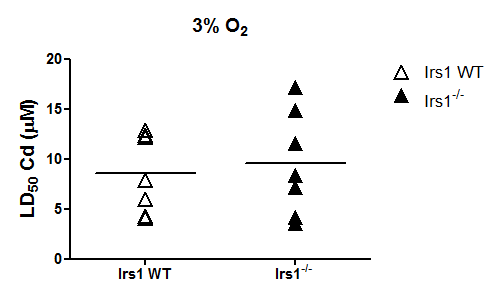

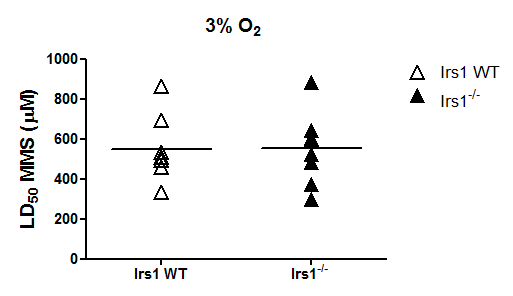

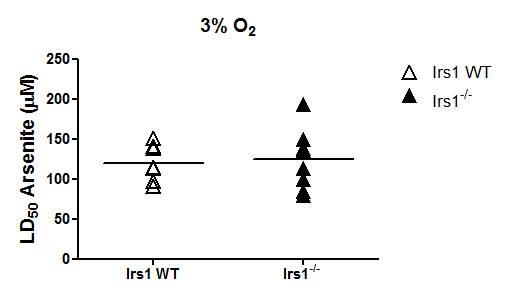


**(A)**

**(B)**

**(C)**

**(D)**

**(E)**

**WT**

***Irs1-/-***

**WT**

***Irs1-/-***

**WT**

***Irs1-/-***

**WT**

***Irs1-/-***

**WT**

***Irs1-/-***

Figure S2

**(A)**

**(C)**

**(D)**

**(B)**

**(E)**

Supplement: Supplementary file 2 — Fig. S1 Fibroblasts from Irs1−/− mice are not more resistant to lethal stress than fibroblasts from WT mice at 3% O2. Fig. S2 Fibroblasts from Irs1−/− mice do not differ in terms of (A) proliferation rates, (B) cell cycle progression or (C) growth rates after 24 h or 48 h of culture (21% O2) compared with cells derived from WT mice (n = 4 per group). [file acel0013-0962-sd2.docx]
