# Supplementary material for: Fibroblasts derived from long-lived insulin receptor substrate 1 null mice are not resistant to multiple forms of stress
Source: Aging Cell. 2014 Jul 24;13(5):962–4. doi: 10.1111/acel.12255 (PMC4331740; doi:10.1111/acel.12255)
Supplement: Supplementary file 3 — Data S1 Experimental Procedures. [file acel0013-0962-sd3.pdf]

Experimental Procedures

Materials

Chemicals were obtained from Sigma Aldrich (Dorset, UK), Fisher Scientific (Leicestershire, UK) and VWR (Leicestershire, UK). Cell culture media, antibiotics and FBS were purchased from VWR (Leicestershire, UK) and Fisher Scientific (Leicestershire, UK). For cell stress experiments, hydrogen peroxide (H_2O_2) was obtained from VWR (Leicestershire, UK and Mississauga, ON, CAN); paraquat (methyl viologen), cadmium chloride, methyl methanesulfonate (MMS) and sodium arsenite were obtained from Sigma Aldrich (Dorset, UK and Oakville, ON, CAN).

Animals

Insulin receptor substrate null (*Irs1*$^{-/-}$) and wild type (WT) mice were caged together in groups of three to six in a temperature controlled room (21 ± 1°C) under a 12L:12D photoperiod (lights out from 1800 to 0600 hours) with a 20 min dawn/dusk phase at either end of the light period. Wood shaving, shredded bedding were provided for enrichment and each cage had cardboard tubes or plastic igloos to offer the mice shelter. Mice had *ad libitum* access to water and standard chow (Special Diets Services (SDS) Rat and Mouse Breeder and Grower Diet; Lillico Biotechnology, Surrey, UK). Genotyping followed previously described protocols (Withers *et al.* 1998; Withers *et al.* 1999). All experiments were carried out following local ethical review, under a licence from the UK Home Office following the ''principles of laboratory animal care'' (NIH Publication No. 86-23, revised 1985). We have previously reported that both male and female *Irs1*$^{-/-}$ mice are long-lived and have relatively improved late-life health (Selman *et al.* 2008;

Selman *et al.* 2011) compared to WT mice, and so both sexes were used in the experiments described here.

Primary dermal fibroblasts cultures

Using WT and *Irs1*$^{-/-}$ mice, whole ears were removed and sterilised in 100% ethanol followed by rinsing with 1 X phosphate buffered saline (PBS, Fisher Scientific, Leicestershire, UK). Ears were digested in Dulbecco's modified Eagle medium (DMEM, high glucose, Thermo Scientific, Leicestershire, UK) supplemented with collagenase type II (final concentration 2mg/ml; Sigma, Dorset, UK) for one hour, with intermittent re-suspension at 37°C in a humidified incubator with 5% carbon dioxide (CO_2) in the air (21% oxygen (O_2)). Digested ears were centrifuged for 10 min at 300 x g and collagenase solution was removed. The pellet was re-suspended on a 35 mm cell culture treated dish (Fisher Scientific, Leicestershire, UK) in fresh complete media (DMEM supplemented with 20% heat-inactivated foetal bovine serum (FBS; Gibco, Leicestershire, UK), antibiotics (100 U/ml penicillin and 100 μg/ml streptomycin; Gibco, Leicestershire, UK) and fungizone (0.25 μg/ml, Gibco, Leicestershire, UK)). Dermal fibroblasts were expanded for one to three passages at 3% O_2 and 5% CO_2 in a CO_2. Cells were split by removing complete media and washing the cells with sterile 1 X PBS, followed by incubating with 1 X trypsin-EDTA (Life Technologies) for approximately 5 min at 37°C in a humidified incubator as above. Trypsin was inactivated with 2 volumes of complete media (Salmon *et al.*, 2005). Confluent cells at the end of the second passage were frozen in heat-inactivated FBS supplemented with 10% dimethyl sulfoxide (DMSO) and stored at -80°C until further use.

Primary myoblast cultures

Isolation of myoblasts from muscle tissue followed a previously published protocol (Robb *et al.* 2012). Briefly, tissue was rinsed in ice-cold sterile 1 X PBS containing penicillin, streptomycin, ampicillin, and gentamicin (Life Technologies). Muscle tissue, under sterile conditions in a biosafety cabinet (Thermo Forma Type II), was minced with a razor and any connective tissue, blood vessels and fat were removed from the muscle. Muscle tissue was rinsed an additional two times in 1 X PBS with the above antibiotics. To each gram of tissue 2 ml of Pronase (1 mg/mL; Sigma) was added and incubated for 90 min at 37°C in a humidified incubator with 5% CO_2, with vigorous trituration every 10 min. Following incubation the suspension was filtered through sterile cheesecloth and centrifuged at 60 x g for 3 min. The supernatant was then centrifuged at 240 x g for 3 min, following this the pellet was washed in sterile 1 X PBS followed by centrifugation at 350 x g for 3 min. The pellet was then resuspended in 10 ml growth media (Ham's/F-10 nutrient mixture containing FBS (Hyclone), non-essential amino acids, basic fibroblast growth factor, penicillin, ampicillin, streptomycin, and gentamycin) and plated onto a collagen coated 10cm^2 diameter tissue culture dish. An additional 10 ml of growth media was added 2 days after plating, and the media was changed 4 days after plating. The outgrowth of myoblasts from tissue explants was typically observed on the fourth day of culture. All myoblast cell lines were expanded for one to three passages at 3% O_2 and 5% CO_2 in a CO_2 incubator (Thermo Forma series II water jacketed). Maintaining 3% O_2 was critical because higher O_2 levels were associated with lower population doubling times and a propensity to differentiate to myotubes (E.L. Robb; pers comm).

Dermal fibroblast stress resistance assays

Dermal fibroblasts oxidative stress resistance assays were performed following previously published protocols (Murakami *et al.* 2003; Salmon *et al.* 2005). Briefly, second passage dermal fibroblasts were incubated at 37°C in a humidified incubator with 5% CO_2 in 3% O_2 and split 3-fold by volume. Third passage cells were counted by hemocytometer and diluted to a concentration of 3 x 10^5 ml^{-1} in complete media supplemented with 10% FBS, antibiotics and fungizone and seeded into a 96-well cell culture-treated microtitre plate at a volume of 100 ml per well and incubated at both 3% and 21% O_2. To assess cellular resistance against the stressors, exposure of the cells to each of the stressors were incubated at 37°C in parallel at 3% and 21% O_2; CO_2 was 5% in the air for both incubators. After approximately 24 h, complete media was removed and the cells were washed with 1 X PBS and incubated at 37°C (3% and 21% O_2) in DMEM supplemented with 2% bovine serum albumin (BSA; Roche Diagnostics, West Sussex, UK), antibiotics and fungizone. Following 24 hours, cells were exposed to a concentration gradient of several cellular stressors; each dose was performed in triplicate. To assess cellular resistance to H_2O_2 (0 – 160 μM), paraquat (0– 5 mM), cadmium chloride (0 – 64 μM), MMS (0 – 600 mM) and sodium arsenite (0 – 400 μM), cells were incubated at 37°C with one of each of the stressors for six h in DMEM. Cell viability was measured 18 h later by the WST-1 test following the manufacturer's protocol (Roche Diagnostics, West Sussex, UK) (Murakami *et al.* 2003; Salmon *et al.* 2005).

Myoblast stress resistance assays

Passage 8 myoblasts were plated onto collagen coated 96-well cell microtitre plates at a density of 5 x 10^5 ml^{-1} at a volume of 200 μl complete media per well and incubated at 3% O_2. After 24 h, complete media was removed and cells were

incubated in non-FBS media for 12 h, after which the media was removed and cell viability was accessed as above following exposure to H_2O_2 (0 – 160 μM) and paraquat (0 – 5 mM). Myoblast stress resistance assays were undertaken at Brock University, St. Catharines, ON, Canada.

Protein extraction from dermal fibroblasts

Confluent dermal fibroblasts were washed with ice cold 1 X PBS followed by incubation in ice cold lysis buffer (10 mM Tris pH 8.0, 150 mM NaCl, 2 mM EDTA, 2 mM dithiothreitol (DTT), 40% glycerol; 0.5% NP40 and protease inhibitor cocktail tablets (Roche) were added to the lysis buffer prior to use) for 15 min. Cell lysates were centrifuged at 13,000 x g (4°C) for 10 min (PerfectSpin; Peqlab). Protein concentration was determined using the Bradford method. Cell lysates were stored at -80°C until use.

Western blotting

Samples were resolved by SDS-PAGE electrophoresis, transferred to PVDF membrane and probed for Nrf2 (1:250; Abcam, Cambridge, UK) Keap1 (1:1000, Abcam). β-actin (1:1000, Cell Signalling) was used as a loading control. Anti-rabbit secondary antibody was obtained from Epitomics (Newmarket, UK) and anti-mouse secondary antibody was obtained from Cell Signalling. Membranes were exposed to chemiluminescence substrate which a digital image was obtained using the Peqlab Fusion SL chemiluminescence camera (Peqlab, Sarisbury Green, UK). Protein bands were analysed with ImageJ software (NIH, Maryland, USA).

RNA extraction and cDNA synthesis

Media was removed from culture dishes and dermal fibroblasts were scraped from the plate with 1 ml TriReagent (Life Technologies, Paisley, UK). RNA was extracted following manufacturer's protocol and the final RNA pellet was re-suspended in RNase free H_2O. The concentration and purity was determined by spectrophotometry (Nanodrop). cDNA synthesis, which followed immediately, was as follows: 2 µg of total RNA was denatured (70°C, 3 min) in the presence of 1µl (500 µg/ml) oligo-dT. The first strand cDNA was synthesized from the total RNA using 1 µl of RevertAid^{TM} reverse transcriptase (10,000 U, Fermentas) in the presence of 5 µl of 5×Reaction Buffer, 1 µl of dNTP (deoxynucleoside triphosphate mix 25mM each) (Bioline), made up to a final volume of 25 µl with H_2O and incubated at 42°C for 2 h.

Real time reverse transcriptase PCR

Real time PCR was performed with a LightCycler 480 (Roche) to quantify the expression of several oxidative stress related genes (Table S3). The primers employed for real time PCR were designed with at least one primer across a predicted intron and pre-tested to ensure that each primer pair could not amplify genomic DNA using the real time PCR protocols. The real time PCRs were performed in duplicate for each sample, along with a 10-fold serial dilution of references consisting of an equimolar mix of purified PCR products of each gene amplified from cDNA. The transcript level was calculated using the quantitative fit points method in the integrated LightCycler 480 software. Fold changes were calculated as the average expression level of each treatment group divided by that of the corresponding controls. The expression of *Gapdh* and *Hprt* genes was used as references.

Dermal fibroblast proliferation and cell cycle assays

Cell proliferation was measured using the CellTrace™ CFSE Cell Proliferation Kit as per the manufacturer's instructions (Life Technologies, Paisley, UK). Third passage cells were counted by haemocytometer and seeded at 1 x 10^4 cells in complete medium supplemented with 10% FBS, antibiotics and fungizone and incubated at 21% O_2. After adherence, cells were incubated with prewarmed PBS containing 10µM CFSE for 15 minutes at 37°C. Subsequently cells were washed and incubated in complete medium. At 24 and 48 hours post-staining, cells were harvested, counted using a haemocytometer with trypan blue and fluorescence was measured using a FACS Canto™ (BD Biosciences). Forward scatter area was used as a measure of cell size. Analysis was performed using FlowJo (Treestar Inc).

To measure cell cycle, cells were harvested after 24 h and 48 h in culture and fixed in 0.4% formaldehyde/PBS for an hour and permeabilised in 0.25% Triton-X-100/PBS overnight at 4^oC. Cells were subsequently washed in PBS and incubated in 5µl of 7-aminoactinomycin (7-aad) antibody (BD Biosciences) and analysed for cell cycle distribution using flow cytometry as above.

Calculation of LD50 values and statistical analyses

To calculate cellular resistance, each cell line (WT and *Irs1*$^{-/-}$) was exposed to each dose of stress in triplicate. LD50, the lethal dose of stress which caused 50% cell death was calculated using probit analysis (SPSS Inc., USA, version 19) (Murakami *et al.* 2003; Salmon *et al.* 2005). Statistical analyses were performed using SPSS (SPSS Inc., USA, version 19) and GraphPad Prism (GraphPad Inc., USA, version 5) software. Data were tested for normality using a Shapiro-Wilk test, normally distributed data was analysed using a general linear model (GLM) with genotype

(WT or *Irs1*$^{-/-}$) and experiment day introduced as fixed factors. All non-significant interaction effects (p>0.05) were removed to obtain the best-fit model in each case. To analyse non-normally distributed data a Kruskal-Wallis test was used. Results are mean ± standard error of the mean (SEM), with p <0.05 regarded as statistically significant. Oxidative stress resistance on myoblasts was completed over a single day; hence a one-way ANOVA was used.

Supplementary references

Murakami S, Salmon A , Miller RA (2003). Multiplex stress resistance in cells from long-lived dwarf mice. *Faseb J.* **17**, 1565-1566.

Robb EL, Maddalena LA, Dunlop VA, Foster T , Stuart JA (2012). Absence of metabolic rate allometry in an ex vivo model of mammalian skeletal muscle. *Comp Biochem Physiol A Mol Integr Physiol.* **162**, 157-162.

Salmon AB, Murakami S, Bartke A, Kopchick J, Yasumura K , Miller RA (2005). Fibroblast cell lines from young adult mice of long-lived mutant strains are resistant to multiple forms of stress. *Am J Physiol Endocrinol Metab.* **289**, E23-29.

Selman C, Lingard S, Choudhury AI, Batterham RL, Claret M, Clements M, Ramadani F, Okkenhaug K, Schuster E, Blanc E, Piper MD, Al-Qassab H, Speakman JR, Carmignac D, Robinson IC, Thornton JM, Gems D, Partridge L , Withers DJ (2008). Evidence for lifespan extension and delayed age-related biomarkers in insulin receptor substrate 1 null mice. *Faseb J.* **22**, 807-818.

Selman C, Partridge L , Withers DJ (2011). Replication of extended lifespan phenotype in mice with deletion of insulin receptor substrate 1. *PLoS One.* **6**, e16144.

Withers DJ, Burks DJ, Towery HH, Altamuro SL, Flint CL , White MF (1999). Irs-2 coordinates Igf-1 receptor-mediated beta-cell development and peripheral insulin signalling. *Nat Genet*. **23**, 32-40.

Withers DJ, Gutierrez JS, Towery H, Burks DJ, Ren JM, Previs S, Zhang Y, Bernal D, Pons S, Shulman GI, Bonner-Weir S , White MF (1998). Disruption of IRS-2 causes type 2 diabetes in mice. *Nature*. **391**, 900-904.
